# The Effect of Experimental Recuperative and Appetitive Post-lunch Nap Opportunities, With or Without Caffeine, on Mood and Reaction Time in Highly Trained Athletes

**DOI:** 10.3389/fpsyg.2021.720493

**Published:** 2021-09-13

**Authors:** Mohamed Romdhani, Nizar Souissi, Ismael Dergaa, Imen Moussa-Chamari, Olfa Abene, Hamdi Chtourou, Zouheir Sahnoun, Tarak Driss, Karim Chamari, Omar Hammouda

**Affiliations:** ^1^Physical Activity, Sport and Health, UR18JS01, National Observatory of Sports, Tunis, Tunisia; ^2^High Institute of Sport and Physical Education, Ksar-Said, Manouba University, Manouba, Tunisia; ^3^PHCC, Primary Health Care Corporation, Doha, Qatar; ^4^College of Education, Physical Education Department, Qatar University, Doha, Qatar; ^5^Regional Center of Sport Medicine, Kairouan, Tunisia; ^6^High Institute of Sport and Physical Education of Sfax, University of Sfax, Sfax, Tunisia; ^7^Laboratory of Pharmacology, Faculty of Medicine, University of Sfax, Sfax, Tunisia; ^8^Interdisciplinary Laboratory in Neurosciences, Physiology and Psychology: Physical Activity, Health and Learning (LINP2), University Paris Nanterre, UFR STAPS, Nanterre, France; ^9^ASPETAR, Qatar Orthopedic and Sports Medicine Hospital, Doha, Qatar; ^10^Research Laboratory, Molecular Bases of Human Pathology, LR19ES13, Faculty of Medicine, University of Sfax, Sfax, Tunisia

**Keywords:** sleep restriction, alertness, psycho-stimulants, midday sleep, cognitive performance

## Abstract

**Purpose:** To investigate the effects of placebo (PLA), 20 min nap opportunity (N20), 5mg·kg^−1^ of caffeine (CAF), and their combination (CAF+N20) on sleepiness, mood and reaction-time after partial sleep deprivation (PSD; 04h30 of time in bed; ***study 1***) or after normal sleep night (NSN; 08h30 of time in bed; ***study 2***).

**Methods:** Twenty-three highly trained athletes (***study 1***; 9 and ***study 2***; 14) performed four test sessions (PLA, CAF, N20 and CAF+N20) in double-blind, counterbalanced and randomized order. Simple (SRT) and two-choice (2CRT) reaction time, subjective sleepiness (ESS) and mood state (POMS) were assessed twice, pre- and post-intervention.

**Results:** SRT was lower (i.e., better performance) during CAF condition after PSD (pre: 336 ± 15 ms *vs*. post: 312 ± 9 ms; *p* < 0.001; *d* = 2.07; Δ% = 7.26) and NSN (pre: 350 ± 39 ms *vs*. post: 323 ± 32 ms; *p* < 0.001; *d* = 0.72; Δ% = 7.71) compared to pre-intervention. N20 decreased 2CRT after PSD (pre: 411 ± 13 ms *vs*. post: 366 ± 20 ms; *p* < 0.001; *d* = 2.89; Δ% = 10.81) and NSN (pre: 418 ± 29 ms *vs*. post: 375 ± 40 ms; *p* < 0.001; *d* = 1.23; Δ% = 10.23). Similarly, 2CRT was shorter during CAF+N20 sessions after PSD (pre: 406 ± 26 ms *vs*. post: 357 ± 17 ms; *p* < 0.001; *d* = 2.17; Δ% = 12.02) and after NSN (pre: 386 ± 33 ms *vs*. post: 352 ± 30 ms; *p* < 0.001; *d* = 1.09; Δ% = 8.68). After PSD, POMS score decreased after CAF (*p* < 0.001; *d* = 2.38; Δ% = 66.97) and CAF+N20 (*p* < 0.001; *d* = 1.68; Δ% = 46.68). However, after NSN, only N20 reduced POMS (*p* < 0.001; *d* = 1.05; Δ% = 78.65) and ESS (*p* < 0.01; *d* = 0.71; Δ% = 19.11).

**Conclusion:** After PSD, all interventions reduced sleepiness and only CAF enhanced mood with or without napping. However, only N20 enhanced mood and reduced sleepiness after NSN. Caffeine ingestion enhanced SRT performance regardless of sleep deprivation. N20, with or without caffeine ingestion, enhanced 2CRT independently of sleep deprivation. This suggests a different mode of action of napping and caffeine on sleepiness, mood and reaction time.

## Introduction

The post-lunch dip (PLD) is characterized by an endogenous increase of sleepiness (e.g., higher sleep propensity and shorter sleep onset latency), favorizing daytime sleep (Monk, 2005; Bes et al., 2009). It is common for athletes to compete or to train during this time of the day (Bes et al., 2009; Romdhani et al., 2019). Therefore, behavioral (i.e., napping) and pharmacological (i.e., caffeine) countermeasure were suggested to enhance alertness during the PLD (Hayashi et al., 2003; Monk, 2005; Waterhouse et al., 2007; Horne et al., 2008; Bes et al., 2009; Romdhani et al., 2020, 2021a,c). Recent evidence suggest that the PLD could be severer after partial sleep deprivation (PSD), yet, these effects were more disturbing after PSD caused by early awakening compared to PSD caused by late bedtime (Romdhani et al., 2019). PSD is common among athletes prior to major competition (Juliff et al., 2015), constantly associated with physical and cognitive performance degradation (Waterhouse et al., 2007; Romdhani et al., 2019, 2020, 2021b). It has been suggested that the decrease in performances maybe secondary to the PSD-induced decreased mood and cognitive functions (Waterhouse et al., 2007; Romdhani et al., 2019, 2021b).

People may take a nap for several reasons. A nap could be a replacement for a lost nocturnal sleep “recuperative nap,” in preparation for a period of sleep loss “prophylactic nap” or even for the joy of napping which refers to “appetitive nap” (Broughton and Mullington, 1992). Those who prefer to nap frequently are called habitual nappers (Milner and Cote, 2009). It has been extensively reported that a short PLD nap enhances cognitive performances and reduces sleepiness when subjects were sleep restricted (i.e., recuperative nap) or well rested (i.e., appetitive nap) (Hayashi et al., 2003; Milner and Cote, 2009; Daaloul et al., 2019; Romdhani et al., 2020, 2021a,c; Souabni et al., 2021). Twenty minutes midday nap has been consistently reported to be the perfect nap duration since it is long enough to produce mood and performance enhancement and short enough to avoid sleep inertia (Hayashi et al., 2003; Milner and Cote, 2009; Souabni et al., 2021). However, comparing the effect of recuperative and appetitive nap on cognitive performances of highly trained athletes remains poorly studied.

On the other hand, caffeine is the most world-wide consumed psychostimulant (McLellan et al., 2016). It is effective in order to offset the sleep loss-induced cognitive performances degradation (Souissi et al., 2014, 2018; Urry and Landolt, 2014; McLellan et al., 2016). However, caffeine's effectiveness may depend on several factors, including but not limited to, the amount of ingested caffeine, habitual caffeine consumption, time of ingestion and the nature of the task (Urry and Landolt, 2014; McLellan et al., 2016). Caffeine effects are dose-dependent with moderate doses (e.g., 5 mg·kg^−1^), ingested 1 h prior to the task, capable of enhancing cognitive performances of sleep deprived athletes, with minimal side effects (McLellan et al., 2016). However, the effects of a caffeine dose may display large inter-individual variability, depending on habitual caffeine consumption (Urry and Landolt, 2014). Indeed, caffeine naïve (<80 mg of caffeine per day) could extract more benefits from the same dose of caffeine compared to heavy caffeine consumers (Bell and McLellan, 2002). Furthermore, it was reported that caffeine has a weak potency to improve executive function impaired by sleep deprivation (McLellan et al., 2016). For instance, it has been reported that 5 mg·kg^−1^ of caffeine mitigated the effect of total (Souissi et al., 2014) and partial (Souissi et al., 2018) sleep deprivation on simple and two-choice reaction time in athletes. With only few studies, the effect of caffeine on cognitive performances after PSD in athletes remains poorly studied (Souissi et al., 2018).

Interestingly, some studies compared the effects of napping to the effects of caffeine (Horne and Reyner, 1996; De Valck et al., 2003; Schweitzer et al., 2006; Horne et al., 2008; Romdhani et al., 2021c). A short mid-afternoon nap was more efficient in reducing sleepiness than a moderate dose of caffeine taken in the early evening (Horne et al., 2008). More interestingly, some studies combined napping and caffeine ingestion and found that the combination of caffeine with a short nap on a wide range of psycho-cognitive tasks (Horne and Reyner, 1996; Reyner and Horne, 1997; Hayashi et al., 2003), and repeated sprint performances (Romdhani et al., 2021c) was better than each of the interventions used separately. For instance, caffeine ingestion immediately prior to a 20 min nap produced better performances compared to caffeine alone or 20 min nap alone (Hayashi et al., 2003). The authors concluded that caffeine prior to 20 min nap mitigated the undesirable effects of sleep inertia at the awakening (Hayashi et al., 2003). However, little is known about the effect of this combination on reaction time and sleepiness of highly trained athletes. Therefore, the current study aimed at investigating the effects of a 20 min nap opportunity (N20), a 5mg·kg^−1^ of caffeine (CAF) or their combination (CAF+N20), as possible countermeasure to the PLD-induced performance decrement after both normal sleep and partial sleep deprivation. For this, reaction time, sleepiness and mood were assessed. Based on the existing literature, we expect that (i) all interventions will enhance cognitive performance and reduce sleepiness, (ii) the combination of the nap and caffeine will result in better performance than each treatment alone, and (iii) the positive effects of these interventions will be lower after normal sleep compared to partial sleep deprivation.

## Methods

### Participants

The sample size was a priori calculated using the G^*^power software (Faul et al., 2007), and following the procedure suggested by Beck (2013). The probability of type I (*p* ≤ 0.05) and Type II (1 – β ≥ 0.95) errors were both fixed at 0.05. Based on an earlier study with a similar paradigm (Romdhani et al., 2019), the effect size (ηp^2^ = 0.52) and correlation between repeated measure (*r* = 0.36) of the main outcome of this study (i.e., simple reaction time) were retained. The G^*^power software indicated a minimal required sample size of 12 participants to achieve an actual power of 0.95.

The first study was conducted during April-May 2015 and the second during April-May 2017. At this period of the year, the day lasted ~13:06 (± 21 min), as the sun rose at 05:46 (± 12 min) and set at 18:52 (± 14 min). Participants in both studies were caffeine naïve [i.e., ≤ 80 mg of caffeine per day (McLellan et al., 2016)], non-habitual nappers, non-smokers and free of drugs. They were all males, highly trained judokas and competing at an international level. Only athletes with moderate and intermediate chronotype were recruited (Horne and Ostberg, 1976). During the month preceding the experiment, sleep diaries were collected and only participants with Pittsburg Sleep Quality Index (Buysse et al., 1989) scores of ≤ 5 were recruited. The present study was conducted in the spirit of the Declaration of Helsinki ethical guidelines (64th World Medical Association General Assembly, Fortaleza, Brazil, October 2013). The local University Institutional Review Board approved the protocols (P-SC N° 009/15). All participants were informed about the study design benefits and risks and signed the informed consent form before the commencement of the assessments. Participants were informed about their right to withdraw from the study at any given time without any penalty.

### Experimental Design (Figure 1)

**Figure 1:**
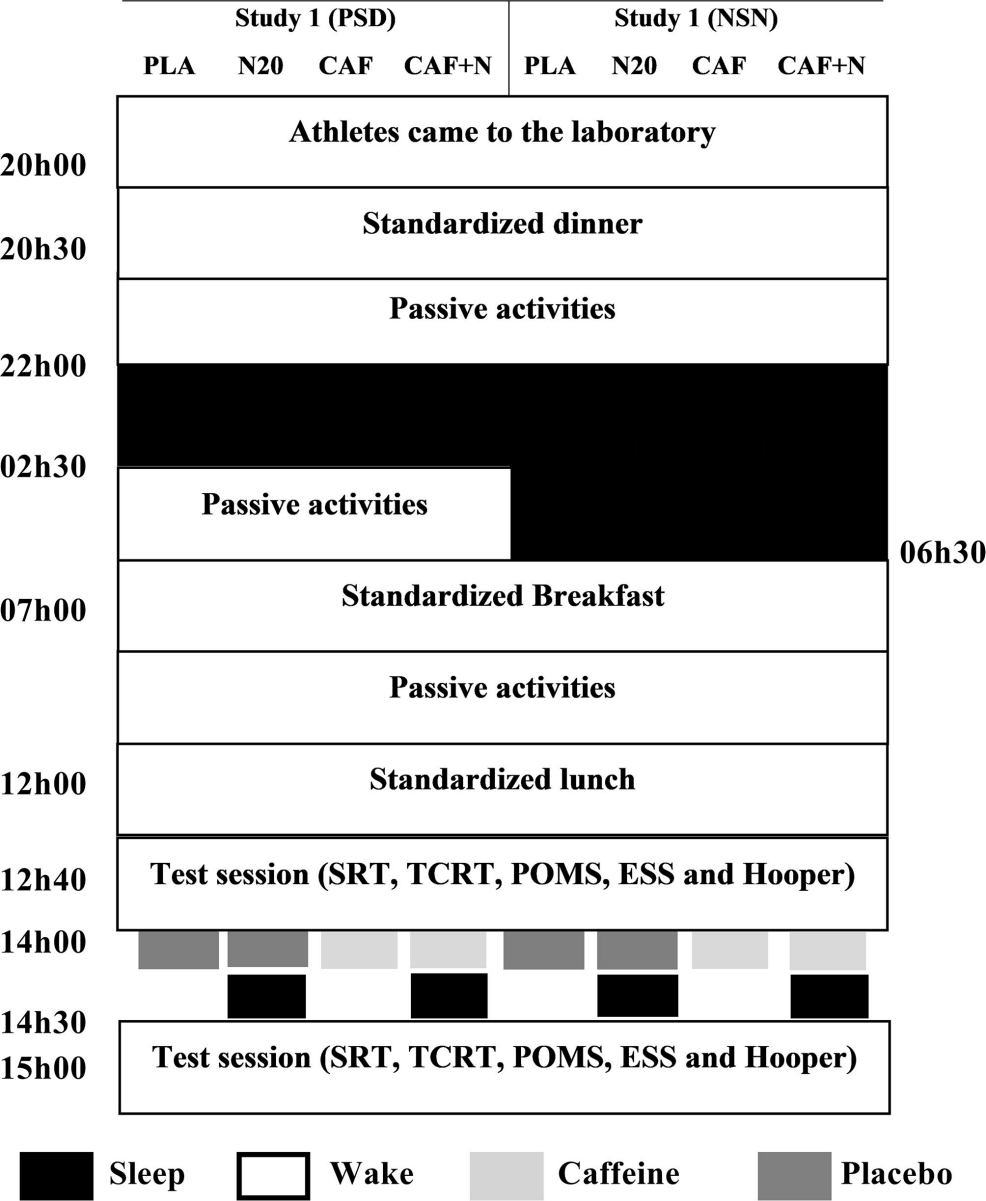
Simplified experimental protocol of the two studies. In the first study (left panel), all sessions were performed after partial sleep deprivation (PSD). In study 2 (right panel), all test sessions were performed after a night of normal sleep (NSN). PLA, placebo ingestion, N20, 20 min nap opportunity with placebo session, CAF, 5mg·kg^−1^ of Caffeine session, CAF+N20, 5 mg·kg^−1^ of Caffeine + 20 min nap session, SRT, Simple reaction time, 2CRT, two-choice reaction time, POMS, profile of mood state, ESS, Epworth sleepiness scale. All times are expressed in local time (GMT + 1 h).

Both studies followed the same protocol; the only difference was the prior night sleep (Figure 1). ***Study 1***; all sessions were performed after a late-night partial sleep deprivation (PSD i.e., time in bed between 22h00 and 02h30). ***Study 2***; all sessions were performed after a normal sleep night (NSN i.e., time in bed between 22h00 and 06h30). During each study, participants accomplished in a counterbalanced, double-blind and randomized order, four test sessions; No-nap with placebo ingestion (PLA), ~20 min nap opportunity with PLA (N20), intake of 5 mg·kg^−1^ of caffeine without napping (CAF) and intake of 5 mg·kg^−1^ of caffeine before N20 (CAF+N20). Sessions were realized at least 1 week apart for washout. During each session, participants came to the laboratory at ~20h00, ate a standardized dinner at ~20h30, followed by ~90 min of free activity (e.g., watching television, playing video-games, surfing on the internet), until 22h00 when they were asked to go bed (all-lights and devices off). Participants were aroused at 02h30 (for PSD nights), and 06h30 (for NSN nights). Then, they ate a qualitatively and quantitatively standardized break-fast at 07h00. They stayed awake until 12h00 doing the same passive activities as in the previous night. During this time, they were asked to not consume food and drank water *ad libitum*. At ~12h00, participants ate a standardized iso-caloric lunch, followed by 40 min of rest. After which simple (SRT) and two-choice (2CRT) reaction time, Profile of Mood State (POMS), wellness Hooper-Index and Epworth Sleepiness Scale (ESS) were administered.

In the napping conditions, participants entered the sleeping room (i.e., comfortably cool, fully dark and quiet) at ~14h00 and ingested a capsule of either (i) 5mg·kg^−1^ of pure powdered-caffeine for CAF+N20 or (ii) cellulose and starch-based placebo for N20. At the same time, participants with the no-nap condition ingested either CAF or PLA. The required amount of caffeine was measured using electronic weighting (± 1 mg) and put into capsules to match placebo in weight, color, and smell. After being permitted to ~10 min to become accustomed to their surroundings, they wore earplugs and eye-masks and the 20 min nap-time started (from 14h10 to 14h30). At the awakening, participants subjectively rated their sleep during the nap opportunity on a 100 mm analog scale; ranging from 0 “no sleep at all” to 100 “deep, uninterrupted sleep.” Participants who failed to initiate sleep (scored 0 at the visual analogue scale) in both studies (4 in *study 1* and 2 in *study 2*) were excluded from the statistical analysis. Post-intervention session started 30 min after the nap to overcome any sleep inertia that might have existed (Waterhouse et al., 2007; Romdhani et al., 2020, 2021a,c). Simple and two-choice reaction times were recorded and questionnaires were filled at ~15h00 in the same pre-nap/rest order. The same period in the PLA and CAF conditions (i.e., from 13h00 to 15h00) was spent watching a neutral documentary seated on a comfortable armchair.

Two participants simultaneously completed the nap condition in separate rooms. They napped in the same room as during the previous night. Participants were instructed to keep the same passive activities during different experimental nights (e.g., watching television, playing video-games, and surfing on the internet). Laboratory conditions were set at: temperature ~ 26°C (± 1.8°C), humidity ~35% (± 3.2%), and luminosity (i) ~200 lux during tests, and (ii) <5 lux during sleep.

### Protocols

#### Simple and Two-Choice Reaction Times

Participants performed simple (SRT) and two-choice (2CRT) reaction times using REACT V0.9 software (Claude Bernard Lyon 1 University, Lyon, France), as described previously (Romdhani et al., 2019, 2020, 2021a).

#### Profile of Mood State (POMS)

POMS standard validated psychological test formulated by McNair (1971) was administered. The questionnaire contains 65 words/adjectives that describe several aspects of mood (i.e., Tension, anger, fatigue, depression, confusion, and vigor). The athlete rates how much he has felt this feeling on a five points Likert-scale (i.e., ranging from 0 “not at all” to 4 “extremely”). The overall POMS score corresponds to the sum of tension + anger + fatigue + depression + confusion sub-scores minus vigor sub-score. The POMS calculator could be found at (***cf***. https://www.brianmac.co.uk/poms.htm). The higher the POMS score is, the lower is the mood state of the athlete.

#### Epworth Sleepiness Scale (ESS)

ESS standard validated questionnaire defines subjective daytime sleepiness (Johns, 1991). The athlete is asked to rate, on a four point Likert-scale (0 “would never dose” to 3 “high chance of dozing”), his/her usual chances of falling asleep while engaged in eight different activities (e.g., sitting and reading, watching TV, and sitting and talking to someone, etc.). Subjective sleepiness scores were correlated with the Multiple Sleep Latency Test, during overnight polysomnography. If the subjective sleepiness score exceeds six, the participant is considered as sleepy (Johns, 1991). For full description of the ESS questionnaire, please see (***cf***. https://epworthsleepinessscale.com/about-the-ess/).

#### Hooper Index Questionnaire

This is a validated psychological self-reporting scale of sleep quality, and fatigue, stress, and delayed onset muscle soreness. Each of these parameters was measured separately using a 7 points subjective rating scales ranging from 1 “very, very low” to 7 “very, very high.” The total score, obtained from the sum of all sub-scales, indicates the athlete's form state or readiness to train (Hooper and Mackinnon, 1995).

### Statistical Analyses

The statistical tests were processed using GraphPad Prism 6 (GraphPad Software, San Diego, CA, USA). All values within the text, figures, and tables are reported as mean ± standard deviation (SD). The Shapiro-Wilks revealed that data were normally distributed, thus parametric tests were used. For each study separately, a One-Way ANOVA with repeated measure was used to assess the difference in subjective sleep quality between sessions (4 interventions). Also, a Two-Way ANOVA with repeated measures was used (4 interventions × 2 timing before and after the intervention) for reaction time and subjective measures. The effect size is reported as eta squared (η^2^) to assess the ANOVA practical significance. Eta-squared values of 0.01, 0.06 and 0.13 represent small, moderate, and large effect sizes, respectively. Once the ANOVA indicated a significant main effect or an interaction, the Bonferroni *post-hoc* test was used. The effect size (*d*) was calculated for pairwise comparison according to Cohen (1992). The magnitude of *d* was classified as to the following thresholds: small (0.2 ≤ *d* < 0.5), moderate (0.5 ≤ *d* < 0.8) and large (*d* ≥ 0.8) (Cohen, 1992). Further, mean difference (*MD*) and the 95% confidence interval (95% CI) are provided for pairwise comparison. Relative delta variation (Δ%) is calculated for pre- to post-intervention comparison to highlight the extent of the intervention effect. The level of significance was set at *p* < 0.05.

## Results

### Participants and Power Analysis

Thirty-three participants were screened to be included in both studies, where four participants failed to fulfill the inclusion criteria (e.g., extreme chronotype, habitual nappers, high caffeine consumers, disturbed sleep or under medication that could interfere with sleep). An overall sample of 29 athletes were involved in both studies and only 23 appropriately completed their corresponding protocol (19.78 ± 1.41 [range 18–22] years, 173.13 ± 7.89 [range 166–192] cm, 71.29 ± 10.02 [range 58–97] kg, BMI = 23.73 ± 2.55 [range 19–33] kg.m^−2^)**.** ***Study 1*** started with 13 athletes; however, only 9 participants appropriately completed the protocol (i.e., see below). For this reason, ***study 2*** started with a higher number of athletes (i.e., 16) and was appropriately completed by 14 athletes.

The achieved power was a *posteriori* calculated and showed an actual power of 0.92 for ***study 1*** and 0.99 for ***study 2***.

### Subjective Sleep Quality

Data of participants who failed to initiate sleep (scored 0 on the visual analog scale) during the nap opportunities was excluded (4 in ***study 1*** and 2 in ***study 2***). Participants reported a higher subjective sleep scores after N20 and CAF+N20 compared to PLA and CAF both after NSN and PSD (all *p* < 0.001), as participants were not allowed to sleep for PLA and CAF conditions. However, subjective sleep during the nap was higher after N20 compared to CAF+N20 following NSN (*p* < 0.001; *d* = 1.12; *MD* = 1.43; 95% CI = 0.42 to 2.43) but not following PSD.

### Simple Reaction Time (SRT)

**Figure 2:**
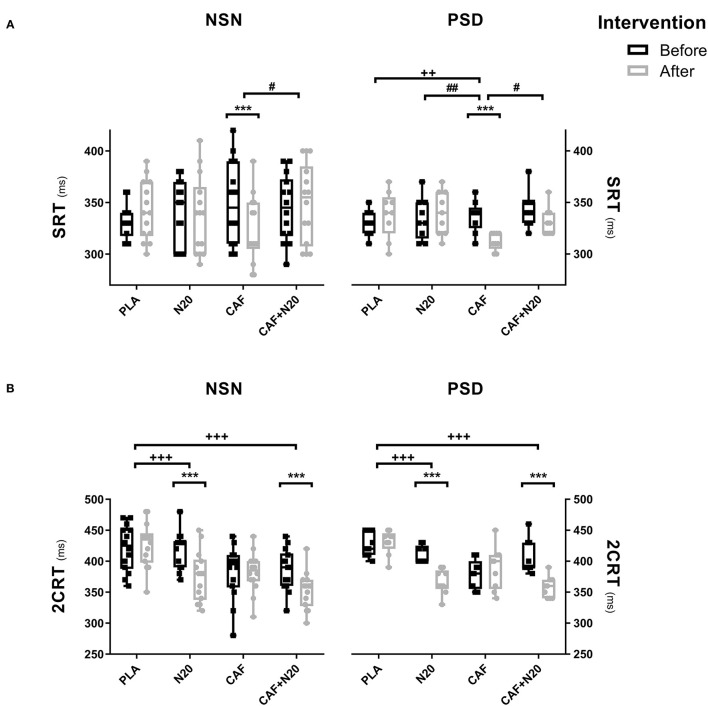
Mean ± standard deviation of simple reaction time (SRT; **A**) and two-choice reaction time (2CRT; **B**) during different protocol sessions; PLA, placebo ingestion, N20, 20 min nap with placebo ingestion, CAF, 5mg·kg^−1^ of caffeine, CAF+N20, 5mg·kg^−1^ of caffeine + 20 min nap after a night of normal sleep (NSN; left panel) partial sleep deprivation (PSD; right panel), ms, milliseconds. Significance was determined using a Two-way repeated measure ANOVA. Significance for pairwise comparison is determined with Bonferroni *post-hoc* test. *, ** and *** presents a significant difference in comparison with pre-intervention values at *p* < 0.05, *p* < 0.01 and *p* < 0.001 respectively, ^+^, ^++^ and ^+++^ presents a significant difference in comparison with PLA values at *p* < 0.05, *p* < 0.01 and *p* < 0.001 respectively, ^#^, ^##^ and ^####^ presents a significant difference in comparison with CAF values at *p* < 0.05, *p* < 0.01 and *p* < 0.001 respectively. μ presents a significant difference in comparison with N20 values at *p* < 0.05.

CAF ingestion reduced (i.e., improve reaction time performance) SRT after PSD compared to pre-intervention (*p* < 0.001; *d* = 2.07; Δ% = 7.26; *MD* = 24.44 ms; 95% CI = 7.61 to 41.28 ms) and compared to PLA, N20 and CAF+N20 (Figure 2A, right panel). Likely, CAF reduced SRT compared to pre-intervention during NSN sessions (*p* < 0.001; *d* = 0.72; Δ% = 7.71; *MD* = 26.04 ms; 95% CI = 6.47 to 46.4 ms) resulting in higher performances compared to CAF+N20 (Figure 2A, left panel). ANOVA's data and pairwise comparison with PLA are presented in Table 1.

**Table 1A:** Simple and two-choice reaction time and self-administered questionnaires' ANOVA output and pairwise comparison with PLA after partial sleep deprivation **(*study 1*)**.

**ANOVA (interaction)**				**PLA vs. N20**				**PLA vs. CAF**				**PLA vs. CAF+N20**			
	***F*_**(3,24)**_**	***p* =**	**η^2^**	**p =**	**d**	**MD**	**95% CI**	***p* =**	**d**	**MD**	**95% CI**	***p =***	**d**	**MD**	**95% CI**
**SRT** (ms)	6.21	0.01	13.6	NS	0.04	1.1	−24.8 to 22.6	0.002	1.57	26.7	8.36 to 44.7	NS	0.43	7.7	15.9 to 31.5
**2CRT** (ms)	11.97	0.001	16.29	0.001	3.33	64.44	38.7 to 90.1	NS	0.49	12.2	−10.4 to 33.9	0.001	4.11	73.33	47.6 to 99.1
**2CRT N°** (au)	6.74	0.001	15.48	0.001	2.01	1.66	0.7 to 2.6	NS	0.21	0.31	−0.6 to 1.2	0.002	2.23	1.33	0.4 to 2.3
**POMS** (au)	21.5	0.001	19.8	NS	0.19	3.55	−10.5 to 3.4	0.001	0.98	7.57	3.3 to 12.2	0.001	0.24	6.11	1.7 to 10.5
**ESS** (au)	24.4	0.001	22.8	0.001	2.03	4.44	2.8 to 6.1	0.001	3.19	5.87	4.3 to 7.4	0.05	1.02	3.22	1.6 to 4.8
**Hooper** (au)	26.22	0.001	24.18	0.001	1.39	1.88	0.6 to 3.1	0.001	1.33	2.16	0.9 to 3.4	0.001	1.7	2.16	0.9 to 3.4

**Table 1B:** Simple and two-choice reaction time and self-administered questionnaires' ANOVA output and pairwise comparison with PLA after normal sleep night (*study 2*).

**ANOVA (interaction)**				**PLA vs. N20**				**PLA vs. CAF**				**PLA vs. CAF+N20**			
	***F*_(3,39)_**	***p =***	**η^2^**	***p =***	**d**	**MD**	**95% CI**	***p =***	**d**	**MD**	**95% CI**	***p =***	**d**	**MD**	**95% CI**
**SRT** (ms)	4.59	0.007	4.71	NS	0.15	5	−29.5 to 39.5	NS	0.62	18.5	−15.9 to 53.1	NS	0.2	7.14	−41.6 to 27.3
**2CRT** (ms)	10.41	0.001	6.05	0.001	1.34	50.7	14.6 to 86.9	NS	0.51	11.2	−8.1 to 30.5	0.001	2.26	73.6	37.4 to 100.1
**2CRT N°** (au)	1.99	NS	3.51	0.002	1.26	1.35	0.4 to 2.3	NS	0.43	0.6	−0.3 to 1.6	NS	0.48	0.7	−0.2 to 1.6
**POMS** (au)	4.41	0.009	5.25	0.002	1.07	9.14	2.6 to 15.7	NS	0.01	0.07	−6.4 to 6.6	0.001	0.52	5.29	−1.24 to 11.8
**ESS** (au)	1.81	NS	3.98	0.006	1.49	1.86	0.4 to 3.3	NS	0.71	1.21	−0.4 to 2.8	NS	0.69	1.25	−0.6 to 3
**Hooper** (au)	1.26	NS	1.08	NS	0.26	0.71	−2.4 to 3.8	NS	0.27	0.85	−2.3 to 4	NS	0.17	0.57	−2.5 to 3.7

*ANOVA, analysis of variance; PLA, Placebo; N20, 20 min nap opportunity; CAF, 5 mg·kg^–1^ of caffeine; CAF+N20, 5 mg·kg^–1^ of caffeine + 20 min nap opportunity; F, F de Fisher; p, probability; η^2^, Eta-squared; d, Cohen's effect size; MD, Mean difference; 95% CI, 95% confidence Interval; NS, Non-significant; SRT, Simple Reaction-Time; ms, millisecond; 2CRT, Two-Choice Reaction-Time; 2CRT N°, number of errors during 2CRT; au, arbitrary unit; POMS, Profile of Mood State; ESS, Epworth Sleepiness Scale*.

### Two Choice Reaction Time (2CRT)

After PSD, 2CRT decreased after N20 (*p* < 0.001; *d* = 2.89; Δ% = 10.81; *MD* = 44.44 ms; 95% CI = 20.33 to 68.56 ms) and CAF+N20 (*p* < 0.001; *d* = 2.17; Δ% = 12.02; *MD* = 48.89 ms; 95% CI = 24.78 to 73.01 ms) compared to pre-intervention values. Also, N20 and CAF+N20 decreased 2CRT compared to PLA (Figure 2B, right panel). Similarly, after NSN, N20 (*p* < 0.001; *d* = 1.23; Δ% = 10.23; *MD* = 42.97 ms; 95% CI = 23.5 to 62.31 ms) and CAF+N20 (*p* < 0.001; *d* = 1.09; Δ% = 8.68; *MD* = 33.6 ms; 95% CI = 14.26 to 53.08 ms) both decreased 2CRT compared to pre-intervention, thus resulting in better performances compared to PLA (Figure 2B, left panel).

The decrease in the number of errors during 2CRT was only significant after N20 both after PSD (*p* = 0.002; *d* = 1.52; Δ% = 60.01; *MD* = 1.33; 95% CI = 0.39 to 2.27) and after NSN (*p* = 0.002; *d* = 1.31; Δ% = 63.55; *MD* = 1.35; 95% CI = 0.42 to 2.29) compared to pre-intervention values (Table 1).

### Epworth Sleepiness Scale (ESS)

**Figure 3:**
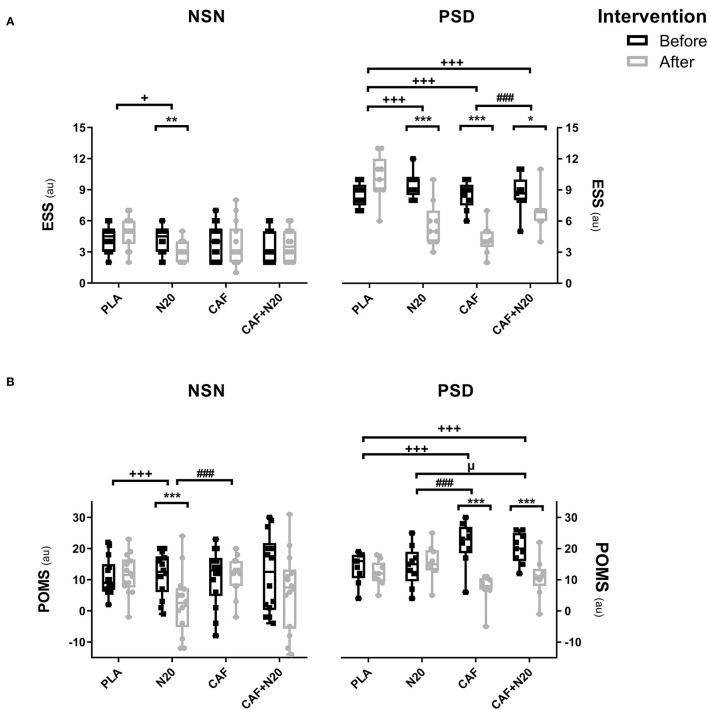
Mean ± standard deviation of Epworth sleepiness scale (ESS; **A**) and profile of mood state (POMS; **B**) during different protocol sessions; PLA, placebo ingestion, N20, 20 min nap with placebo ingestion, CAF, 5mg·kg^−1^ of caffeine, CAF+N20, 5mg·kg^−1^ of caffeine + 20 min nap after a night of normal sleep (NSN; left panel) partial sleep deprivation (PSD; right panel). Significance was determined using a Two-way repeated measure ANOVA. Significance for pairwise comparison is determined with Bonferroni *post-hoc* test. *, ** and *** presents a significant difference in comparison with pre-intervention values at *p* < 0.05, *p* < 0.01 and *p* < 0.001 respectively, ^+^, ^++^ and ^+++^ presents a significant difference in comparison with PLA values at *p* < 0.05, *p* < 0.01 and *p* < 0.001 respectively, ^#^, ^##^ and ^###^ presents a significant difference in comparison with CAF values at *p* < 0.05, *p* < 0.01 and *p* < 0.001 respectively, ^μ^, ^μμ^ and ^μμμ^ presents a significant difference in comparison with N20 values at *p* < 0.05, *p* < 0.01 and *p* < 0.001 respectively.

After PSD, ESS score was lower after CAF ingestion (*p* < 0.001; *d* = 3.14; Δ% = 50.11; *MD* = 4.25; 95% CI = 2.45 to 6.06), N20 (*p* < 0.001; *d* = 2.18; Δ% = 40.01; *MD* = 3.77; 95% CI = 1.97 to 5.57) and after CAF+N20 (*p* < 0.05; *d* = 0.69 Δ% = 20.17; *MD* = 1.74; 95% CI = 0.26 to 3.21) compared to pre-intervention values (Table 1). Also, ESS score was lower after all interventions compared to PLA and after CAF compared to CAF+N20 (Figure 3A, right panel). During NSN sessions, only N20 decreased ESS score compared to pre-nap (*p* < 0.01; *d* = 0.71; Δ% = 19.11; *MD* = 0.86; 95% CI = −0.59 to 2.23) and to PLA (Figure 3A, left panel).

### The Profile of Mood State (POMS)

After PSD, POMS score decreased (i.e., mood improved) after CAF (*p* < 0.001; *d* = 2.38; Δ% = 66.97; *MD* = 14.57; 95% CI = 10.47 to 18.68) and CAF+N20 (*p* < 0.001; *d* = 1.68; Δ% = 46.68; *MD* = 9.38; 95% CI = 5.28 to 13.51) compared to pre-intervention scores (Figure 3B). POMS scores were lower after CAF and compared to PLA and N20 (Figure 3B, right panel). During NSN sessions, POMS score was lower only after N20 (*p* < 0.001; *d* = 1.05; Δ% = 78.65; *MD* = 9.21; 95% CI = 2.41 to 16.01) compared to pre-intervention (Figure 3B, left panel).

### Hooper Wellness Questionnaire

After PSD, all interventions enhanced (i.e., lower Hooper score) wellness; N20 (*p* < 0.001; *d* = 2.28; Δ% = 26.31; *MD* = 2.77; 95% CI = 1.37 to 4.18), CAF (*p* < 0.001; *d* = 2.1; Δ% = 36.21; *MD* = 4.25; 95% CI = 2.85 to 5.66) and CAF+N20 (*p* = 0.009; *d* = 1.38; Δ% = 18.84; *MD* = 1.74; 95% CI = 0.33 to 3.14) compared to pre-intervention. However, after NSN, all interventions had non-significant effect on wellness score (Table 1).

## Discussion

The present study compared the effect of experimental appetitive and recuperative nap opportunity with or without caffeine ingestion on mood, sleepiness and reaction time in highly trained athletes. This study showed that all interventions enhanced mood and reduced sleepiness after partial sleep deprivation (PSD) but not after normal sleep night (NSN), partially accepting the first hypothesis. The combination of caffeine and napping (CAF+N20) was not any better than a moderate dose of caffeine alone (CAF) or a 20 min nap opportunity alone (N20) on reaction time, rejecting our second hypothesis. Finally, the impact of these interventions on subjective measurements (mood and sleepiness) was greater after PSD compared to NSN, but their effects on reaction time were similar after both PSD and NSN, partially accepting the third hypothesis.

SRT decreased (***i.e.***, better performance) only after CAF ingestion, regardless of sleep deprivation status. SRT is the duration that separates the emergence of a simple/single stimulus and its' subsequent response (Burke et al., 2017). Thus, caffeine ingestion enhanced what was not enhanced by a short nap (i.e., produced a faster stimulus detection and/or response execution), even when the two interventions were combined together. Otherwise, 2CRT was shorter whenever participants napped regardless of caffeine ingestion and/or sleep deprivation. CAF had no effect on 2CRT. Even when consumed before the nap, caffeine ingestion did not result in further enhancement on 2CRT. Thus, better 2CRT performance refers only to napping and not to caffeine ingestion. 2CRT differs from SRT, as it is composed of stimulus identification, response selection/programming and thereafter, response execution (Schmidt and Wrisberg, 2008). Immediately after the recognition of the stimulus, the two responses which are in their initial activation stage begin a competition, and the execution starts when one of the responses reaches a pre-defined threshold (Coles et al., 1985). A plausible explanation implies that the short nap, unlike caffeine ingestion that enhances stimulus detection and/or response execution, enhanced the correctness of the stimulus identification and response selection. It could be possible that napping enhanced the neuronal signaling in the responsible brain areas, which are believed to be the anterior cingulate cortex and the supplementary motor area (Burle et al., 2004). Confirming this speculation, the number of correct answers during 2CRT increased only during N20 sessions after PSD and NSN.

All interventions enhanced wellness and mood-state and reduced sleepiness compared to PLA after PSD, with caffeine having the greatest impact compared to napping. Similarly, it has been reported that both caffeine and a short nap restored performances and reduced sleepiness to the same levels, with caffeine being slightly better (Horne and Reyner, 1996; Reyner and Horne, 1997; De Valck et al., 2003; Philip et al., 2006; Sagaspe et al., 2007). This was true only after PSD, because only N20 produced a better mood and lower sleepiness compared to PLA after NSN, being in line with earlier studies (Hayashi et al., 2003; Romdhani et al., 2021a). This could be explained by the fact that pre-intervention sleepiness was higher and subjective mood state was lower after PSD (***study 1***) compared to NSN (***study 2***). Thus, CAF reduced pre-intervention sleepiness after PSD which was high, and the already low sleepiness after NSN may require a higher dose of caffeine, in line with the Yerkes-Dodson inverted U-shape hypothesis (McLellan et al., 2016). Another plausible explanation is that CAF was more efficient than N20 because of the duration of wakefulness at the moment of testing. In fact, athletes spent longer duration since wake during ***study 1*** (12 h 30 min) compared to ***study 2*** (8 h 30 min). Taken together, the current results favor a short nap over a moderate dose of caffeine, at least for enhancing mood and reducing sleepiness both after NSN and PSD.

Speaking of the effect of the caffeine and nap combination (CAF+N20), our findings indicate that the combination was not any better than each treatment alone on subjective and objective measurements, aligning with earlier reports (Bonnet and Arand, 1994; Horne and Reyner, 1996; Reyner and Horne, 1997; Hayashi et al., 2003; Schweitzer et al., 2006). However, other studies showed a greater performance after CAF+N20 compared to caffeine alone or napping alone (Bonnet and Arand, 1994; Horne and Reyner, 1996; Reyner and Horne, 1997; Hayashi et al., 2003; Schweitzer et al., 2006; Romdhani et al., 2021c). The participants' characteristics could explain these discrepancies. First, participants in the current study were non-habitual nappers. It has been reported that non-habitual nappers display a heavier sleep inertia upon awakening (Milner and Cote, 2009). Moreover, our participants were caffeine naïve. An earlier study (Bell and McLellan, 2002) reported that compared to heavy caffeine consumers, caffeine naïve athletes showed a greater performance enhancement after the same dose used in the current study (i.e., 5mg·kg^−1^).

The potential ergogenic effect of napping and caffeine were essentially discussed in occupational settings, such as night shift (Bonnet and Arand, 1994; De Valck et al., 2003; Schweitzer et al., 2006) and long car driving (Horne and Reyner, 1996; Reyner and Horne, 1997; Philip et al., 2006; Sagaspe et al., 2007). What is innovative in this study is the targeted population (i.e., highly trained athletes). 2CRT assesses flexible thinking in a pressing setting of reaction time (e.g., including the ability to cancel a pre-planned action and replace it with another), which is highly required in open-skill sports. Judo, like other combat or team sports, is an open skill sport that requires a high level of attention and cognitive readiness (Romdhani et al., 2019). Major competitions in Judo (major tournaments, world championships and Olympic Games) entail the whole day where preliminaries took place in the morning and finals in the afternoon separated by a lunch break. Clearly, the short post-lunch nap enhanced flexible thinking, with or without caffeine consumption, after both sleep deprivation and normal sleep. Therefore, a 20 min nap opportunity during the post-lunch dip (lunch break) could be of use to enhance flexible thinking in the subsequent competition (e.g., during the finals). On the other hand, caffeine ingestion enhanced SRT (i.e., stimulus detection and/or response execution), both after sleep deprivation and normal sleep. Although the difference was not that huge (i.e., 24 milliseconds after PSD and 26 milliseconds after NSN), significant faster stimulus detection could make a difference in performance (and potentially on the podium) during 100 meters race or 50 meters swimming, for instance (Pain and Hibbs, 2007; Abbes et al., 2021). Therefore, a moderate dose of caffeine could be of use for athletes competing at closed skill (all out) sports discipline.

## Strengths and Limitations

The main strength of the study is that the protocol reflects real-life settings (i.e., where athletes are free from any attached device that could interfere with sleep quality) and the study assumptions could be readily used in such conditions. However, the actual sleep duration during the nap opportunity was subjectively rated, which could be a limitation, because sleep onset latency presents variability due to the habitual experience with daytime sleep, prior nocturnal sleep and the time of day. The used paradigm presents a reliable laboratory tool in assessing reaction time; however, it does not reflect the real sport field settings. For instance, the actual sleep duration during laboratory night was not recorded. Further, the small sample size of study one could limit to some extent the relevance of our findings. Besides, no female athletes were recruited in the current study. Thus, future studies with more controlled paradigms are warranted to confirm the actual results with more adapted sport-specific tests, larger sample size and on female athletes. The current conclusions should be treated with cautions, taking into account that our participants were highly trained, non-habitual nappers and caffeine naïve athletes. It could be possible that the ergogenic effect of caffeine observed in this study resulted from athletes being caffeine naïve. Thus, extrapolation of the current conclusions in a regular caffeine consumer cohort may require higher caffeine doses.

## Conclusions

For subjective mood and sleepiness, a moderate dose of caffeine could be better than a short nap after partial sleep deprivation. However, after normal sleep, the short nap resulted in better mood and lower sleepiness compared to caffeine. When athletes are performing a task that requires frequent decision making, a short nap could be better than a moderate dose of caffeine both after normal sleep or sleep deprivation. Nevertheless, when performing a simple reaction time tasks, caffeine ingestion could lead to better results compared to napping. The combination of caffeine and a short nap was not any better than each intervention used separately.

## Data Availability Statement

The raw data supporting the conclusions of this article will be made available by the authors, without undue reservation.

## Ethics Statement

The studies involving human participants were reviewed and approved by the University of Manouba Institutional Review Board (P-SC N° 009/15). The patients/participants provided their written informed consent to participate in this study.

## Author Contributions

MR, NS, and OH conceived and designed the study. MR, OA, ZS, and ID collected the data. MR analyzed the data. MR, ID, and IM-C drafted the manuscript. TD, HC, and KC critically revised the manuscript. MR takes responsibility for the integrity of data and the accuracy of the data analysis. OH is the guarantor for the work and/or conduct of the study. All authors approved of the final version to be published and agree to be accountable for any part of the work.

## Funding

No external funding related to the project has been received. Open Access funding is provided by the Qatar National Library.

## Conflict of Interest

The authors declare that the research was conducted in the absence of any commercial or financial relationships that could be construed as a potential conflict of interest.

## Publisher's Note

All claims expressed in this article are solely those of the authors and do not necessarily represent those of their affiliated organizations, or those of the publisher, the editors and the reviewers. Any product that may be evaluated in this article, or claim that may be made by its manufacturer, is not guaranteed or endorsed by the publisher.
